# Infusoria in Woman’s Milk

**Published:** 1854-04

**Authors:** 


Infusoria in Woman's Milk.—By. Dr. Vogel.—No general directions can be given as to whether a woman may suckle or not. In every case the question must be determined by an examination of the milk; and here the microscope proves eminently useful. The author found in that milk which produced sickness in the child, and destroyed the health of the mother after prolonged lactation, immediately after its removal from the breast, infusoria similar to those found in the incrustations upon the teeth (*vibrio bacillus*). Such vibriones are found especially in women who menstruate or suffer from hœmorrhages during this period, the good or bad aspect giving no important indication. The milk has often a fine, thick, white color, or is of paler hue; its consistence may be either thick or watery; its re-action is often alkaline, but generally neutral. Under the microscope it exhibits, according to its richness, sometimes but few, at other times many, milk and cream corpuscles; these differ from the corpuscles of healthy milk by their pale yellow color, their want of metallic lustre, and their speedy decomposition. As regards the infusoria, they are little rod-shaped bodies, dark in the middle, surrounded by a lighter line, but exhibiting neither head nor tail under a magnifying power of 600 diameters; there are, however, feet in great number and of considerable length. The movement of these animalcules was swimming, and occasionally it was very active. Forward movement was worm-like, and an annular stricture of four rings was observed. Mostly they twist screw-like, upon their axes. When they swim in a circle, they always move from right to left. The length is one-hundredth mmtr., their breadth four times less. They are best seen when the milk is diluted with water. In ammonia-diluted acids (even the lactic) they die immediately.

Children fed upon milk containing these infusoria, become sooner or later attacked by diarrhœa, and the evacuations are of a green color. This condition disappears as soon as healthy cow's milk is substituted. The author believes that this effect does not proceed from the infusoria as such, but from the same cause which produces the infusoria, namely, a process of fermentation in the milk itself. The ferment is, according to him, the congested and increased heat in the breasts, connected with the general excitement of the sexual system.

But a fermentation, as Jul. Clarus observes, cannot be present, because the author always found the milk alkaline or neutral, and never sour. Were there fermentation, the evolution of lactic acid would, upon the author's own showing, have immediately destroyed the infusoria.—*Schmidt's Jahrb.*

